# Enhancing synthetic lethality of PARP-inhibitor and cisplatin in BRCA-proficient tumour cells with hyperthermia

**DOI:** 10.18632/oncotarget.15922

**Published:** 2017-03-06

**Authors:** Arlene L. Oei, Caspar M. van Leeuwen, Vidhula R. Ahire, Hans M. Rodermond, Rosemarie ten Cate, Anneke M. Westermann, Lukas J.A. Stalpers, Johannes Crezee, H. Petra Kok, Przemek M. Krawczyk, Roland Kanaar, Nicolaas A.P. Franken

**Affiliations:** ^1^ Laboratory for Experimental Oncology and Radiobiology (LEXOR), Center for Experimental Molecular Medicine, Academic Medical Center (AMC), Amsterdam, The Netherlands; ^2^ Department of Radiotherapy, Academic Medical Center (AMC), Amsterdam, The Netherlands; ^3^ Department of Medical Oncology, Academic Medical Center (AMC), Amsterdam, The Netherlands; ^4^ Department of Cell Biology and Histology, Academic Medical Center, Amsterdam, The Netherlands; ^5^ Department of Molecular Genetics, Cancer Genomics Center Netherlands, Utrecht, The Netherlands; ^6^ Department of Radiation Oncology, Erasmus University Rotterdam (EUR), Rotterdam, The Netherlands

**Keywords:** PARP1-inhibitor, hyperthermia, synthetic lethality, cDDP

## Abstract

**Background:**

Poly-(ADP-ribose)-polymerase1 (PARP1) is involved in repair of DNA single strand breaks. PARP1-inhibitors (PARP1-*i*) cause an accumulation of DNA double strand breaks, which are generally repaired by homologous recombination (HR). Therefore, cancer cells harboring HR deficiencies are exceptionally sensitive to PARP1-*i*. For patients with HR-proficient tumors, HR can be temporarily inhibited by hyperthermia, thereby inducing synthetic lethal conditions in every tumor type. Since cisplatin is successfully used combined with hyperthermia (thermochemotherapy), we investigated the effectiveness of combining PARP1-*i* with thermochemotherapy.

**Results:**

The *in vitro* data demonstrate a decreased in cell survival after addition of PARP1-*i* to thermochemotherapy, which can be explained by increased DNA damage induction and less DSB repair. These *in vitro* findings are in line with *in vivo* model, in which a decreased tumor growth is observed upon addition of PARP1-*i*.

**Materials and Methods:**

Survival of three HR-proficient cell lines after cisplatin, hyperthermia and/or PARP1-*i* was studied. Cell cycle analyses, quantification of γ-H2AX foci and apoptotic assays were performed to understand these survival data. The effects of treatments were further evaluated by monitoring tumor responses in an *in vivo* rat model.

**Conclusions:**

Our results in HR-proficient cell lines suggest that PARP1-*i* combined with thermochemotherapy can be a promising clinical approach for all tumors independent of HR status.

## INTRODUCTION

Many cancer treatment modalities either interfere with DNA metabolism or with the repair of DNA damage. Combination of different, carefully selected modalities can enhance clinical outcomes. Here, we investigate the effectiveness of different combinations of three modalities which interfere with DNA integrity or DNA repair: cisplatin (cDDP), mild hyperthermia (heating the tumour to 40–43°C for 1 h) and Poly-(ADP-ribose)-polymerase1 inhibitors (PARP1-*i*).

A high percentage of breast and ovarian cancers (~50–85% and ~30–66 %, respectively) are caused by mutations in *BRCA1* or *BRCA2* genes [1–4]. Tumours with mutations in either of these genes require homologous recombination (HR) for repair [5]. Inactive HR can be due to mutations in BRCA1 or BRCA2, which may result in potentially lethal accumulation of DNA double strand breaks (DSBs). HR-deficient (c.q. BRCA-deficient) cells are thus exquisitely sensitive to PARP1-*i* [6]. Importantly, this also implies that healthy, HR-proficient cells are not targeted by PARP1-*i*, which makes this therapy particularly desirable for patients with HR-deficient tumours [5, 7]. Clinical trials have indeed confirmed the effectiveness of PARP1-*i* as a single treatment against BRCA-deficient tumours [8, 9].

In HR-proficient tumours, synthetic lethality can also be induced by combining PARP1-*i* with a local treatment of mild hyperthermia [5, 6, 10–15], which causes degradation of BRCA2 for several hours [13] and thereby HR deficiency at the heated tumour site. Combination of hyperthermia (HT) with PARP1-*i* thus creates a possibility to induce synthetic lethality in every tumour type that can be heated locally [13, 16]. Cisplatin (cDDP) is a widely used chemotherapeutic agent that is combined with HT (hence called thermochemotherapy) as standard treatment for previously irradiated patients with recurrent cervical a. behind [17–19] cDDP induces DSBs that are usually repaired by HR, because cDDP disrupts the non-homologous end joining (NHEJ), the other major DSB repair pathway [20, 21]. In absence of HR and NHEJ, a PARP1-dependent back-up NHEJ (b-NHEJ) pathway can take over the repair of DSBs [22]. As a consequence, a combination of HT, cDDP and PARP1-*i* could potentially cause an overload of DSBs while simultaneously interfering with all major DSB repair pathways [23]. The accumulation of unrepaired DSBs can result in cell death.

In this study, HR-proficient cell lines (R1, SiHa, HeLa) and a HR-proficient rhabdomyosarcoma allograft model were used to investigate the effectiveness of treatments combining PARP1-*i*, with mild HT and cDDP. Cell survival as well as cell cycle analyses, quantification of γ-H2AX foci and apoptotic assays were performed. Finally, the effects of the different treatments on tumour outgrowth were measured *in vivo*.

## RESULTS

### Addition of PARP1-i to cDDP-based thermochemotherapy diminishes cell survival

**Figure 1:**
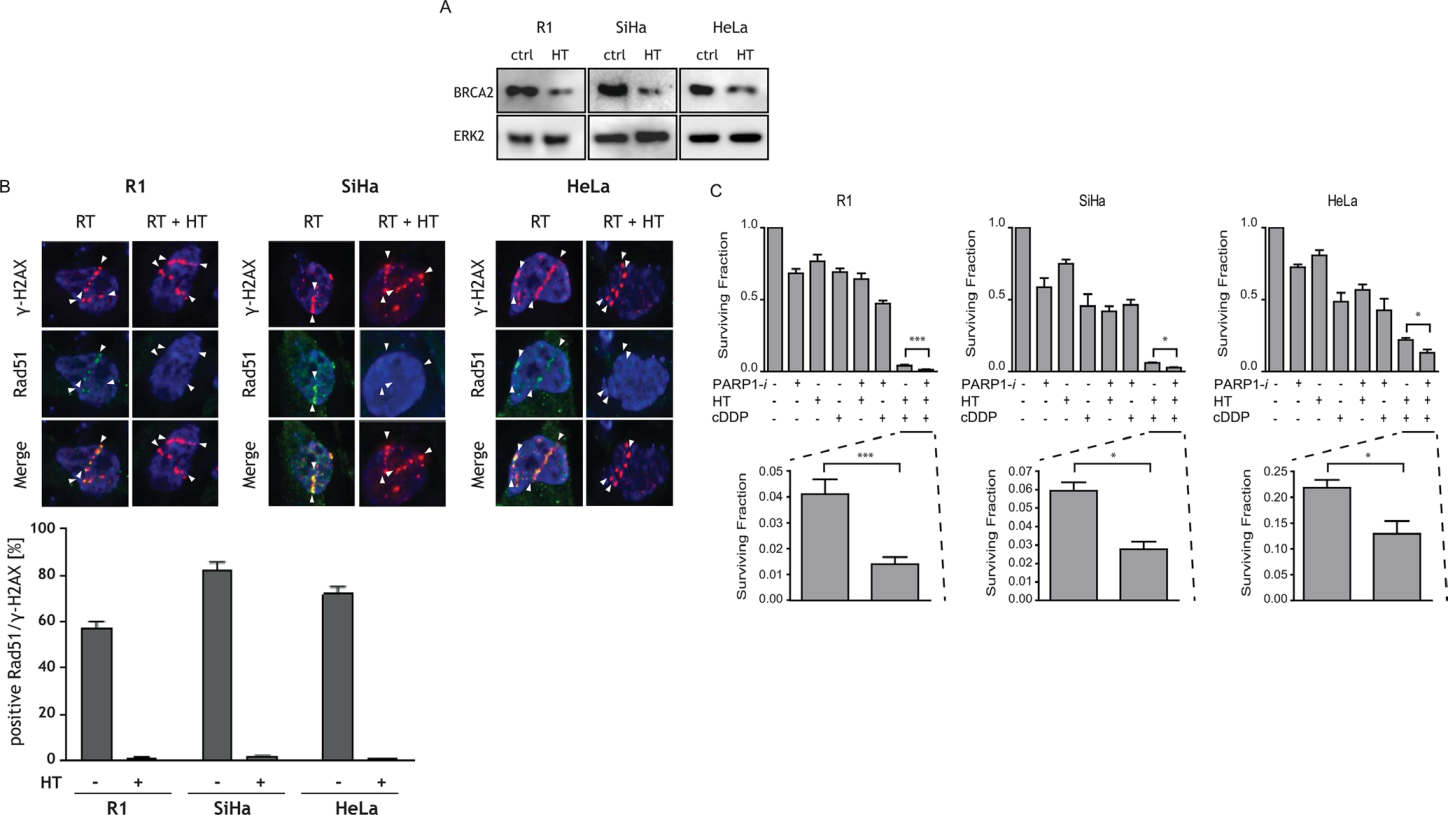
The effects of PARP1-*i*, HT, cDDP and combined treatments on BRCA2, Rad51 and cell survival are shown (**A**) Western blots demonstrating R1, SiHa and HeLa cells are BRCA2 proficient. After HT, BRCA2 is downregulated. (**B**) γ-H2AX and Rad51 co-localization, to investigate activity of homologous recombination. (**C**) Clonogenic assays were performed to study the effect of the different treatment combinations, 10–12 days after treatments. The addition of PARP1-*i* to cDDP-based thermochemotherapy resulted in a significantly lower cell survival compared to cDDP-based thermochemotherapy alone. R1: *p* = 0.0008, SiHa: *p* = 0.034, HeLa: *p* = 0.021. The bar graph shows the mean of at least five independent experiments. From left to right: R1, SiHa, Hela cells. *p < 0.05, **p < 0.01, ***p < 0.001.

The response of cells to HT was investigated for BRCA2 levels (Figure 1A). After HT, BRCA2 levels are downregulated. Effects of HT on homologous recombination were studied by scoring co-localization of Rad51 and γ-H2AX (Figure 1B). After RT alone, a clear co-localization of γ-H2AX, a protein detecting DNA double strand breaks and Rad51, a protein involved in homologous recombination is observed. After RT + HT, Rad51 foci are absent, indicating homologous recombination is not active anymore. To directly evaluate the effect of combinations of treatments with HT, cDDP and PARP1-*i*, clonogenic assays were conducted using the R1, SiHa and HeLa cells (Figure 1C). For all cell lines, therapy with the PARP1-*i* alone killed 30–40% of the cells. As such, treatment with PARP1-*i* was only slightly more effective than HT as a single treatment. cDDP was the most effective monotherapy. The combination treatment of PARP1-*i* with HT was equally effective as cDDP alone, and more effective than PARP1-*i* or HT alone. PARP1-*i* combined with cDDP was more effective than cDDP alone in the R1 cell line. In SiHa and HeLa cells, PARP1-*i* plus cDDP demonstrated a small decrease in cell survival, compared to cDDP alone. Combinational treatment of cDDP and HT was highly toxic and around 80–90% of the cells did not survive this treatment.

The addition of PARP1-*i* to cDDP-based thermochemotherapy caused a higher than 2-fold reduction in cell survival in R1 cells, an almost 2-fold reduction in SiHa cells and a ~1.5-fold reduction in HeLa cells.

### Triple modality treatment leads to accumulation of DNA damage

**Figure 2:**
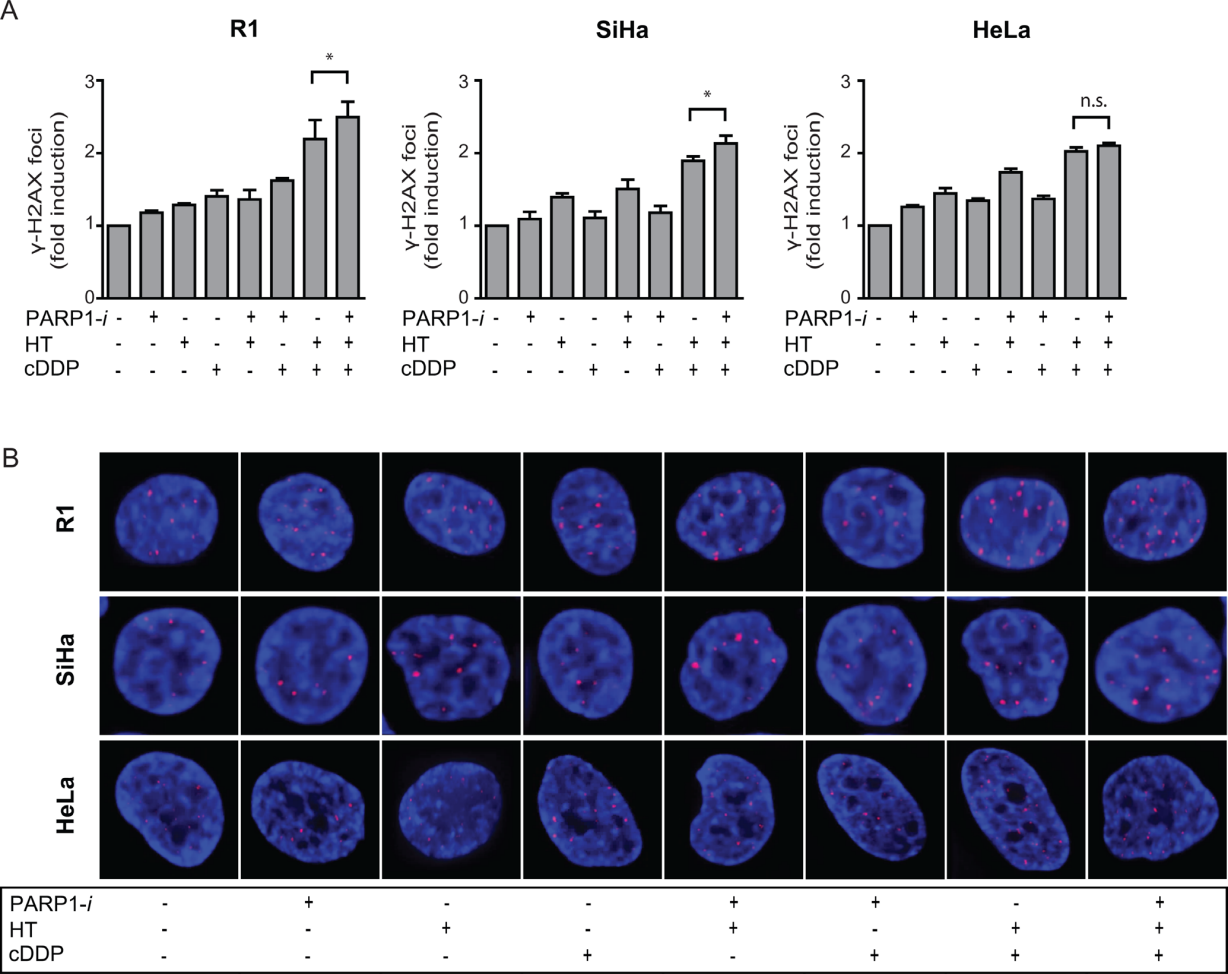
DSBs were analysed using the γ-H2AX assay (**A**) The induction of DSBs in R1 and SiHa was significantly higher after addition of PARP1-*i* to cDDP-based thermochermotherapy. In HeLa cells this was not found to be significant, although a trend is seen. R1: *p* = 0.048, SiHa: *p* = 0.035, HeLa: *p* = 0.068 From left to right: R1, SiHa, Hela cells. (**B**) One representative cell is depicted for each condition. Bars represent the mean of three independent experiments with the standard error of the mean (SEM). *p < 0.05.

Formation of γ-H2AX, which represents unrepaired DSBs, was analysed by flow cytometry, in order to identify a possible mechanism for differences in cell survival analyses after the triple modality treatment (Figure 2A). Cells grown on cover slips, treated with different combinations of cDDP, HT and PARP1-i were used for immunocytochemistry. For each condition one representative cell is depicted in Figure 2B. An up to 1.5-fold increase in γ-H2AX intensity was found after any of the single- and double-treatments. The load of DNA damage after addition of PARP1-*i* to cDDP-based thermochemotherapy was significantly higher than after cDDP-based thermochemotherapy alone.

### Triple modality treatment increases the fraction of cells in S-phase

**Figure 3:**
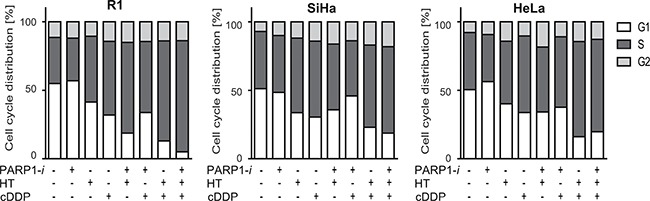
Cell cycle distribution using BrdU incorporation While the monotherapies hardly ever changed the cell cycle analyses, any treatment containing cDDP resulted in a larger proportion of cells in S-phase. The differences found after adding PARP1-*i* to thermochemotherapy were not significant. From left to right: R1, SiHa, Hela cells.

Cell cycle distribution was studied by incorporation of BrdU. In the untreated samples, ~50% of R1, SiHa and HeLa cells were in G1-phase, ~40% in S-phase and ~10% in G2-phase of the cell cycle (Figure 3). Treatment with PARP1-*i* caused modest changes in cell cycle distribution, while after HT a slight decrease in G1 cells was observed, combined with a minor increase of cells in the G2-phase. Of all monotherapies, cDDP had the strongest effects on cell cycle distribution in R1 cells, causing increased fraction of S-phase cells, as compared to the control. Moreover, in all cell lines, an increased proportion of cells in S-phase was found after a single treatment with cDDP and hyperthermia and after the triple modality.

### Addition of PARP1-i to cDDP and hyperthermia increases apoptosis

**Figure 4:**
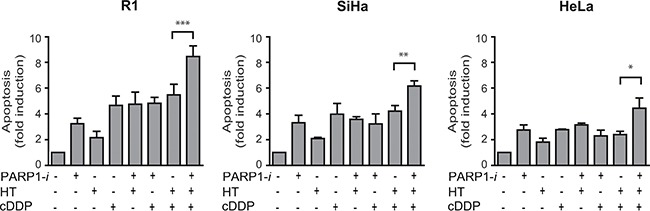
Apoptotic levels measured using the Nicoletti assay An ~1.5-fold induction in apoptosis is found after treatment with PARP1-*i*, cDDP and HT compare to cDDP with HT in R1 and SiHa cells (R1: *p* = 0.0007, SiHa: *p* = 0.0054). A smaller, though significant effect was found in HeLa cells after addition of a PARP1-*i* (HeLa: *p* = 0.026). From left to right: R1, SiHa, Hela cells. *p < 0.05, **p < 0.01, ***p < 0.001.

To examine whether cell death detected in the clonogenic assays is due to induction of apoptosis, a Nicoletti assay was performed. The results of this assay demonstrate a 2 to 5-fold induction in apoptotic levels in R1 cells after any combination of treatment, except after the triple modality, for which there was an almost 9-fold induction of apoptosis (Figure 4). The degree of induction differed per cell line, but the trend was similar for all tested cell lines.

### The triple modality treatment delays tumour outgrowth *in vivo*

**Figure 5:**
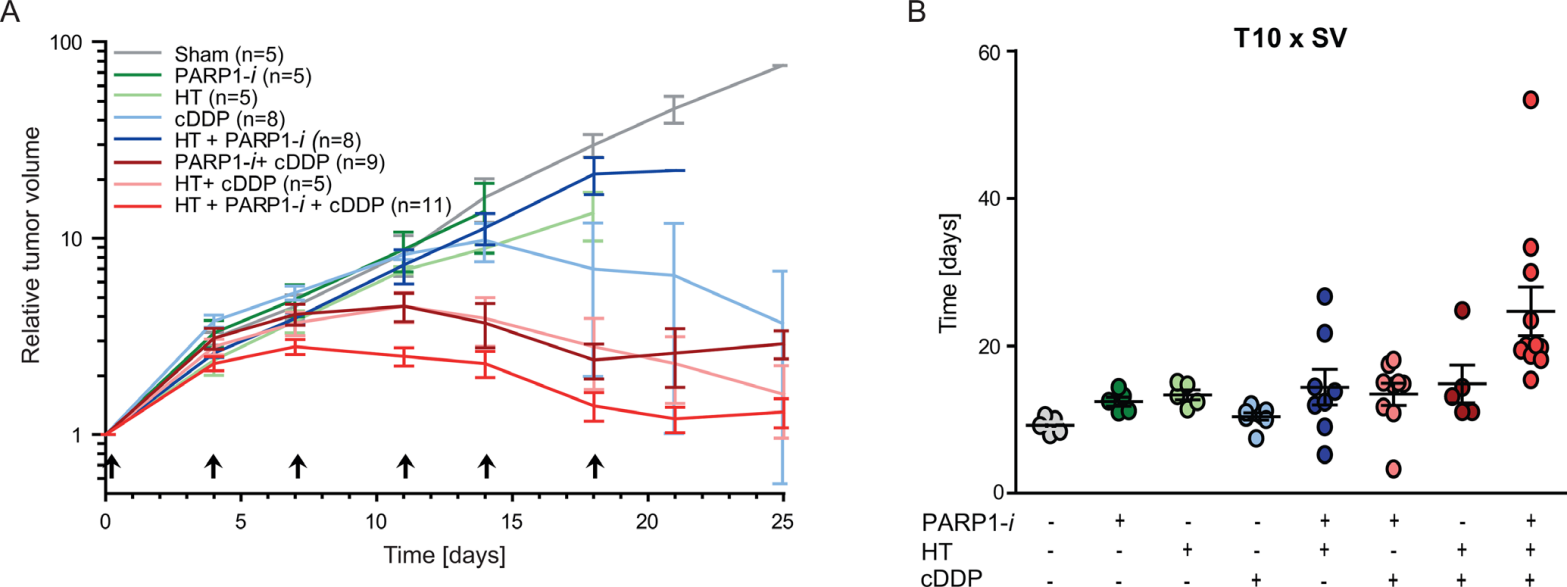
*In vivo* experiments performed on rats (**A**) Relative tumour volume demonstrated that PARP1-*i* enhanced the effectiveness of cDDP-based thermochemotherapy and reduced the *in vivo* tumour outgrowth. Arrows present the time of treatment. (**B**) Time needed to achieve ten times the start volume (T10 × SV). Animals treated with the triple modality showed a delay in reaching T10 × SV compared to thermochemotherapy or any single treatment. All animal experiments included at least five rats per group.

*In vivo*, tumours were treated with any combination of PARP1-*i*, HT and cDDP to test whether the therapy induces similar effects *in vivo* as *in vitro*. In untreated rats the tumour volume increased approximately exponentially, without a significant change in the slope of the growth curve. A treatment with cDDP alone, cDDP combined with either PARP1-*i* or HT, or cDDP combined with both induced a change in the steepness of the curve (Figure 5A), indicating that these treatments contributed to tumour control. Effects on tumour growth delay range from modest in the groups treated with PARP1-*i*, HT and HT+PARP1-*i* to substantial after cDDP alone, PARP1-*i* + cDDP, PARP1-*i* combined with HT and the triple modality. The differences in tumour growth were also calculated as the period of time needed to reach 10 times the tumour starting volume (T10 × SV) (Figure 5B). While the mono-therapies had only marginal influence on reaching T10 × SV, compared to the untreated animals, the double treatments did cause a delay of a few days. The highest level of tumour control was seen after the triple modality treatment, where the tumours took, on average, almost twice as long to reach the T10 × SV, as compared to the single therapies and approximately 1.5 times longer compared to cDDP-based thermochemotherapy. Importantly, no (acute) toxicities were detected in any of the rats.

## DISCUSSION AND CONCLUSIONS

Our data demonstrated that addition of PARP1-*i* to cDDP-based thermochemotherapy results in an almost 2-fold reduction in cell survival, as compared to cDDP-based thermochemotherapy alone. This can be explained in part by an increased induction of apoptosis, which might be due to the higher DNA damage load, as indicated by γ-H2AX analysis. This, in turn, could result in increased activation of the S/G2 checkpoint, as we observed a higher fraction of cells in S-phase after cDDP-based thermochemotherapy and an even more profound increase after the triple modality treatment. Prolonged cell cycle block could eventually lead to apoptosis. It is important to stress that all these effects were caused by a single treatment session and we expect that a repeated treatment regimen, more akin to clinical settings, would lead to stronger responses.

After the last treatment the tumour growth recovered. Although the double treatment with HT+cDDP and PARP1-*i*+cDDP were almost as effective in controlling tumour growth (Figure 5A), in terms of reaching ten times the start volume, the triple modality considerably delays tumour growth.

Clinical trials that have been performed on patient with BRCA-deficient tumours received daily Olaparib, sometimes even twice a day [8]. However, in our study, we mimic the BRCA-deficiency by applying HT. This results in a temporary downregulation of BRCA and within this timeframe, the PARP1-*i* should be administered. Therefore, the animals in our study were treated twice a week with two times Olaparib and one dose of cisplatin on the day of HT treatment. Thus, although it is not an identical schedule to the previous clinical trials with Olaparib, it is the most optimal scheme for treating BRCA-proficient tumours with HT and PARP1-*i*. As a consequence, since we have not treated daily, and only for a period of three weeks, it may be a valid consideration to extend the treatments with a couple of weeks, as HT sessions in patients are normally given once or twice a week for a period of five weeks in our center. Therefore, the outcomes of this study are promising, because we have treated these tumours with just 60% of the normal HT scheme and already the tumour shrank to its starting volume. However, an additional agent to standard thermochemotherapy may increase toxicity levels. Whether this occurs should be investigated.

There is growing evidence that platinum-based agents can also contribute in interfering with HR, resulting in HR-deficiency [24]. Subsequently, it might be interesting to combine platinum-based agents with PARP1-*i*. However, some people state that the use of PARP1-*i* may be limited in the clinic due to an increase in observed toxicities in combination with chemotherapeutic agents [25, 26]. The triple modality that we propose, PARP1-*i* combined with cDDP-based thermochemotherapy might have more potential, because HT can make chemotherapeutic agents penetrate more deeply into the tumour and HT targets hypoxic areas, which are generally least sensitive to other anti-cancer therapies. Thus, lower chemotherapeutic dose may be as effective as normal concentrations without HT, but at a reduced toxicity [16].

In summary, there have been many studies that use the HR-deficiency in BRCA-mutated tumours to induce potentially lethal DNA damages by inhibition of PARP1. The advantage of our study is the possibility to treat BRCA-proficient cells with an already successful clinically applied therapy that can temporarily inhibit the HR. Therefore, we may induce synthetic lethality for any tumour type.

## MATERIALS AND METHODS

### Cell lines and cell culture

Rat rhabdomyosarcoma cells (R1, a cell line developed in our own institute for *in vitro* cultures as well as for *in vivo* growing tumors [27]) were grown in MEM. The cervical carcinoma cells (SiHa and HeLa) were obtained from the American Type Culture Collection (ATCC) and grown in EMEM. All cell lines are BRCAwt. All media contained 25 mM Hepes (Gibco-BRL life technologies, Breda, The Netherlands) supplemented with 10% heat-inactivated foetal bovine serum (FBS) and 2 mM glutamine. Cells were maintained in a 37°C incubator with humidified air supplemented with 2% and 5% CO_2_, respectively. The cell division time of R1 cells was approximately 16 h and of SiHa and HeLa cells 24 h.

### *In vitro* treatments

Cells were treated for approximately 60 min with 5 μM cisplatin (cDDP; Platosin^®^, Pharmachemie B.V., Haarlem, The Netherlands) and/or continuously with 100 μM of PARP1-*i* (dissolved in DMSO; NU1025, Tocris Bioscience, Bristol, UK). cDDP was added 5 minutes before HT, while PARP1-*i* was added 30 min prior to the HT treatment. Immediately after HT, medium was refreshed and PARP1-*i* was re-added until the end of the experiment.

HT was performed by partially submerging the culture dishes in a thermostatically controlled water bath (Lauda aqualine AL12, Beun de Ronde, Abcoude, The Netherlands) for 1 h at 42°C. In order to check the temperature, thermocouples were placed in parallel culture dishes; the desired temperature (± 0.1°C) was reached in approximately 5 min. HT was performed in an atmosphere containing 2% (R1 cells) or 5% (SiHa and HeLa cells) CO_2_ with gas mixture inflow rate of 2 L/min.

### Western blotting

To identify the BRCA2 status of these cell lines, western blots were conducted. Also, the response to HT was tested. Cells were harvested 30 min post treatment. Next, pellets were lysed on ice in ice-cold RIPA buffer (20 mM Tris-HCl, 150 mM NaCl, 1 mM Na_2_EDTA, 1% NP-40, 1% sodium deoxycholate, 2.5 mM sodium pyrophosphate, 1 mM beta-glycerophosphate, 1 mM Na_3_VO_4_, 1 μg/ml leupeptin) with protein inhibitors for 30 min [28]. Laemmli buffer containing fresh added 2-mercaptoethanol (355 mM) was mixtued to the supernatant (1:1), before heated in boiling water for 2–5 min. Finally, samples were sonificated (Sonics & Materials Inc). One μg of protein was loaded on 4–15% SDS-PAGE gradient precast gels (BioRad) and transferred to PVDF membranes. Loading of protein were checked by Ponceau S staining. Immunodetection was performed for anti-BRCA2 (antibodies-online) and anti-ERK2 (Bethyl Laboratories), and a secondary anti-rabbit (Invitrogen Life Technologies). All samples were enhanced using chemoluminescence (Amersham Pharmacia Biotech). Eventually, blots were analysed using LAS4000 (GE, Healthcare life sciences).

### Co-localization of Rad51 and γ-H2AX foci

Activity of homologous recombination has been investigated by co-localization of Rad51 and γ-H2AX foci. Cells were plated on 1.8 μm mylar membrane dishes. One day later, cells were treated with or without HT prior to a min irradiation of α-particles. Afterwards, 30 min after treatment, cells were fixed with 2% paraformaldehyde. Cells were washed with PBS and permeabilized during a 30-minute incubation with TNBS (PBS containing 0.1% Triton X-100 and 1% FCS), before staining with γ-H2AX (Millipore, dilution 1:100 in TNBS) and Rad51 antibody (dilution 1:25 in TNBS) as described by Bergs *et al* [29]. Eventually, a drop of vectashield containing DAPI (Life technologies, USA) was pipetted in the slide and the coverslip is turned upside down on the slide. Samples were scored under the fluorescence microscope.

### Cell survival assay

Cell survival was studied for R1, SiHa and HeLa cells using different combinations of HT, cDDP and PARP1-*i*. Clonogenic assays were conducted as described by Franken et al. [30]. Cells were plated at 4 h prior to treatment into 6-well culture plates (Costar, USA). Dishes were placed in a 37°C incubator with the desired percentages of CO_2_ at 37°C until sufficiently large clones were formed, which took approximately 10–12 days. Afterwards, the medium was removed and cells were washed with PBS before a 30-min incubation in a 2–3 ml of a mixture containing 6.0% glutaraldehyde and 0.5% crystal violet, at room temperature. Finally, plates were washed with tap water and dried at room temperature. Colonies were counted under a light microscope [31]. Surviving fractions were calculated by dividing the plating efficiency of treated cells by that of control cells [32].

### Detection of DNA DSBs quantifying γ-H2AX foci and flow cytometric detection of γ-H2AX

To identify a possible mechanism for the differences in cell survival after the triple modality treatment, we quantified the formation of γ-H2AX, representing unrepaired DSBs, by flow cytometry. For immunocytochemistry, to present a representative image, cells were plated 24 h before any combination of cDDP, PARP1-*i* and HT treatment on sterile coverslips. On the next day, cells were fixed for 10 min in PBS containing 2% paraformaldehyde, three times washed with PBS. Then, cells were permeabilised during a 30-min incubation in TNBS (PBS containing 0.1% Triton X-100 and 1% FCS). Afterwards, cells were stained for 90 min at room temperature with 50 μl primary antibody of a mouse monoclonal anti-γ-H2AX (Millipore, dilution 1:100 in TNBS). Cells were washed once in PBS and two times in TNBS before staining for 30 min at room temperature with a secondary antibody anti-mouse Cy3 (Jackson, diluted 1:100 in TNBS). After three times washing with TNBS, vectashield-containing DAPI (Life technologies, USA) was dropped onto glass slides, before placing the coverslips upside-down on the slides. γ-H2AX foci were scored under a fluorescence microscope.

To quantify γ-H2AX using flow cytometry, the staining was performed at 24 h after treatment. Cells were centrifuged (1200 rpm, 10 min), prior to fixation in a mixture containing 2 ml PBS and 6 ml absolute ethanol. Next day, cells were washed and incubated on ice for 10 min with PBS + 0.1% triton and 4% BSA. After centrifugation, cells were stained with antibodies against γ-H2AX-FITC (4 μg/ml, Merck Millipore, USA). Samples were then analysed using FACS Canto (BD Biosciences, USA).

### Cell cycle analysis

The thymidine analogue 5-Bromo-2′-deoxy-uridine (BrdU, Sigma Aldrich, USA) was used to analyse cell cycle distribution. At 16 h after treatments, BrdU (diluted 1:100) was added for 60 min at 37°C, before fixating cells in a mixture containing 2 ml PBS and 6 ml of absolute ethanol. Cells were stored at 4°C. The next day, after spinning down samples at 1200 rpm for 2 min, pellets were resuspended in pepsin-HCl (0.4 mg/ml, 0.1 N HCl) and cells were incubated for 30 min at room temperature. Cells were then washed with PBT (PBS containing 0.5% Tween-20 (Sigma Aldrich USA)). After centrifugation, pellets were resuspended in HCl (2 N, Merck) and incubated for 30 min at 37°C. Next, cells were stained with rat-anti-BrdU primary antibody (Abcam, UK) diluted 1:100 in PBTb (1% bovine serum albumin, Sigma, in PBT) for 60 min at 37°C. After washing cells two times with PBT and once with PBTg (PBT containing 1% normal goat serum (Dako, USA), cells were stained with a goat-anti-rat secondary antibody labelled with FITC (Abcam, UK) diluted 1:100 in PBTg for 60 min at 37°C. Cells were washed three times with PBS before resuspending the pellets in PBS. Finally, propidium iodide (Sigma-Aldrich, USA) was added and cell suspensions were vortexed. Analysis was performed using the FACS Canto flow cytometer (BD Biosciences, USA).

### Apoptosis assay

To study apoptosis after any combination of treatments, the Nicoletti assay [30] was performed. Cells were collected 48 h after the different treatments and pellets were resuspended in Nicoletti buffer (0.1% w/v Sodium citrate, 0.1% v/v Triton X-100 in demi water, pH 7.4), containing propidium iodide (Sigma-Aldrich, USA). Analyses were performed using the FACS Canto flow cytometer (BD Biosciences, USA).

### Animal experiments

Female WAG-Rij rats were implanted with 1 mm^3^ piece of rhabdomyosarcoma tumour tissue in the right hind leg. After approximately three weeks, when the tumours had grown to 50–100 mm^3^, the animals were treated twice a week for a period of 3 weeks. The treatment involved HT for 1.5 h at 42°C in a thermostatically controlled water bath. In order to monitor the temperature thermocouples were placed next to the tumours. Earlier experiments [13, 34] demonstrated that when the water bath was set to 42.7°C, the intra-tumour temperature was 42°C. Animals were cooled to prevent them from receiving whole body hyperthermia. The PARP1 inhibitor Olaparib was dissolved in a mixture of 10% 2-hydroxy-propyl-β-cyclodextrin and 10% DMSO in PBS and administrated at a dosage of 50 mg/kg p.o. 16 h and 2 h prior to HT. cDDP (Platosin^®^, Pharmachemie B.V., Haarlem, The Netherlands) was given i.p. at the dosage of 2.0 mg/kg 2 h prior to hyperthermia. Animal weight was monitored every two days and tumour sizes were measured using a Vernier caliper every two days, until the end of the experiment. Animals were sacrificed when the tumour volume exceeded 3500 mm^3^. The treated hind leg of rats was checked every two days for skin irritation. All animal experiments included at least five rats per group.

### Statistical analyses

All *in vitro* data represent means (of at least three experiments) with standard error of the mean (SEM) of at least three independent experiments. SPSS (Chicago, IL, USA) statistical software using a non-parametric Mann-Whitney test was used to analyse cell survival, γ-H2AX foci and apoptosis. *P*-values lower than 0.05 were considered statistically significant.
